# Skin Temperature of Slaughter Pigs With Tail Lesions

**DOI:** 10.3389/fvets.2020.00198

**Published:** 2020-04-30

**Authors:** Dayane Lemos Teixeira, Laura Ann Boyle, Daniel Enríquez-Hidalgo

**Affiliations:** ^1^Instituto de Ciencias Agroalimentarias, Animales y Ambientales (ICA3), Universidad de O'Higgins, San Fernando, Chile; ^2^Departamento de Ciencias Animales, Pontificia Universidad Católica de Chile, Santiago, Chile; ^3^Teagasc Animal and Grassland Research and Innovation Centre, Fermoy, Ireland; ^4^Bristol Veterinary School, University of Bristol, Langford, United Kingdom

**Keywords:** skin temperature, tail lesions, pigs, tail biting, casualty

## Abstract

The aim of this study was to assess the effect of tail lesion severity on skin temperature of slaughter pigs measured at the base of the tail and the ear by infrared thermography camera and to evaluate the association between the temperature measurements. Pigs were randomly selected in the lairage, containing ~200 pigs/pen. Tail lesions were scored according to severity, using a 0-4 scale. Tail lesion scores 0 and 1 were summed as it was difficult to distinguish healed lesions from mild lesions due to animal dirtiness. In total, 269 study pigs were imaged at the two locations. The effect of tail lesion score and sex of the pig on the highest temperature of the infrared image areas were analyzed using linear mixed models. Association between the tail base and ear base temperatures was evaluated using Pearson correlation. Skin temperature measured at the base of the tail was significantly lower for tails scored 0–1 than for all other tail lesion scores (*P* < 0.05). Pigs with tail lesion scored 2 had significantly lower skin temperatures at the base of the tail than pigs with tail lesion scored 3 or 4 (*P* < 0.05) while there was no difference in skin temperature at the base of the tail between pigs with tail lesion scored 3 and 4 (*P* > 0.05). Skin temperature measured at the ear base was significantly lower for pigs with tail lesion scored 0–1 than pigs of all other tail lesion scores (*P* < 0.05) with no difference between the other scores (*P* > 0.05). Furthermore, there was an association between the two measurements (*r* = 0.50; *P* < 0.001). The findings suggest that even pigs with moderate tail lesions (score 2) may have general inflammation and infection, evidenced by the elevated systemic temperature compared to pigs with none or mild tail lesion (score 0–1).

## Introduction

Tail biting is a widespread damaging behavior performed by pigs which causes major welfare problems in intensive pig production systems (1, 2). As an endemic, multifactorial phenomenon (3) it is difficult to understand the specific on-farm factors that are responsible for the behavior (4). Tail biting is linked to impaired welfare in the biter because it reflects disharmony between the pig and its environment (5). Being a victim is stressful (6, 7) and painful (7, 8) and in extreme cases leads to death (1). Tail bitten pigs are reluctant to spend time feeding to avoid exposing the tail (9), and thus reduce feed intake and average daily gain, resulting in lower carcass weight (1, 7, 10–12).

Extensive investigations into the epidemiology and consequences of tail biting (11, 13, 14) show that it also reflects deficiencies in the pigs' health status (15). Tail lesions are associated with inflammation and changes in stress physiology (7), and provide a route for infection (8, 9, 16, 17) that can be hematogenously disseminated to different organs (8, 9, 18). Tail lesions present three separate routes for dissemination of infection around the body with local, in the form of pelvic abscessation or osteomyelitis, or systemic consequences: pyemia or multiple abscessation of distant anatomical locations (9, 17).

Tail lesions are associated with certain pathological lesions (3, 9, 19–22), particularly with carcass abscessation (12, 18, 20, 21, 23), but also with viscera and carcass condemnation (22, 24). Furthermore, welfare-related lesions, such as tail damage (9, 25), are associated with chronic stress (e.g., hypocortisolism) (7), which suppresses the immune system. Therefore, aside from providing an entry point for pathology, tail lesions can also contribute to an increased prevalence and duration of disease (26, 27), such as gastric lesions (28), and respiratory organ inflammation and disease (6).

The body surface temperature is determined by the heat exchange between the skin and environment, the metabolic activity and the blood circulation in the anatomical structure close to the body surface (29). The normal body temperature in resting pigs varies between 37.0 and 39.6°C (30), but under stressful situations it can increase up to 41.0°C (31). Previous studies have attempted to measure the skin temperature of pigs as an indicative measure of their body temperature (32, 33), especially using the ear base as a reference (34).

Infrared thermography cameras provide fairly accurate temperature measurements with good spatial and thermal resolution being very convenient in veterinary medicine. Infrared thermography is in use in different studies aiming to improve detection of unhealthy animals, including early identification of clinical symptoms (34, 35) and fever (36) or high temperature (>39.5°C) detection (29). Infrared thermography has the advantage over other temperature measurement methods used in veterinary medicine as it is not invasive and avoids spreading infection among sick animals. Furthermore, infrared thermography images display the distribution of surface temperature, allowing the analysis of (hot or cold) spots in selected areas for subsequent detection of possible anomalies (34). Infrared thermography images are suitable for measuring skin temperature in pigs, as the density of their hair offers several bare skinned surface areas, especially at the ear base and the udder (34).

The aim of this study was to assess the effect of tail lesion severity on skin temperature of slaughter pigs measured at the base of the tail and the ear by infrared thermography camera, and to evaluate the association between the temperature measurements. Due to systemic inflammation and/or infection, we hypothesized that increasing severity of tail lesion would be associated with increased skin temperature in the regions.

## Materials and Methods

### Data Collection

The study was conducted in four experimental days during April and May 2019, with similar weather conditions, in a Chilean abattoir. Data were collected where the pigs (Large White x Landrace; 100–110 Kg of body weight) were lairaged in pens at the abattoir before slaughtering (water was available but no food). Pigs were mixed prior to transport but not after arrival at the abattoir. All data were collected between 10 am and 3 pm. Data were collected from 269 study pigs. A total of 55% of the study pigs were castrated males. All pigs were tail docked (3-5 cm of length). Pigs were randomly selected in the lairage, containing ~200 pigs/lairage pen. The origin of pigs (farm/batch), number of lairage pens and the period that they remained in the lairage were not recorded.

**Figure 1:**
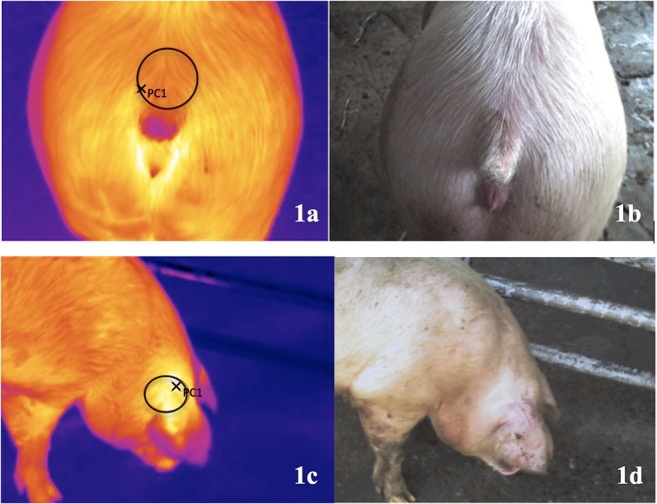
Digital thermography images (DTI) from the base of the tail **(a)** and the ear **(c)**; digital image real time recorded parallel to each thermal image through a built-in digital camera [tail base: **(b)**; ear base: **(d)**]. Data were collected where the pigs were lairaged in pens at the abattoir before slaughtering.

Throughout the study, the same person scored tail lesions and subsequently took a digital thermography image (DTI) and a digital image was real time recorded parallel to each thermal image through a built-in digital camera (TESTO 875-2i; Entech Industrial Solution Co., Ltd.; Thailand). The area of the tail region selected was the loin area adjacent to the base of the tail (Figures 1a,b). The ear base area was selected to be imaged (Figures 1c,d) as it is considered one of the best skin locations due to its high correlation with pig's rectal temperature (34). The tail lesion score, the sex of the pig (female or castrated male) and the number (code) of the images were registered by a second person, who also marked the pigs on the lumbar area using animal marking sticks (Raidex) to track animals already measured for the study. Both observers entered into the pen by its gate and moved smoothly through the pigs.

**Figure 2:**
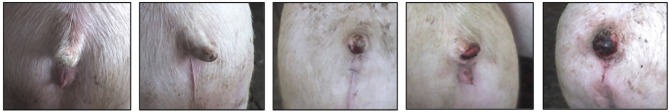
Tail lesion scoring system adapted from Kritas and Morrison (20) and Harley et al. (37). (Scores 0–4, left to right). (0) No evidence of tail biting—clean, white unblemished tail; (1) Superficial disruption of the skin of the tail tip and/or light to darker brown marks along the length of the tail but no obvious evidence of teeth marks/puncture wounds on the tail; no swelling, no necrotic tissue; (2) Evidence of teeth marks (puncture wounds) on the tail tip and/or length; characterized by disrupted epidermis and redness but no evidence of swelling; (3) Evidence of teeth marks/puncture wounds (chewing); disrupted epidermis with redness and swelling but no necrotic tissue; (4) Open wound on the tail or where tail used to be; redness, swelling and necrotic tissue evidenced by yellowish (pus) slough or eschar (dry, black, hard necrotic tissue).

Tail lesions were scored according to severity. The 0-4 scale (Figure 2) was adapted from Kritas and Morrison (20) and Harley et al. (37). As pigs were dirty and laired in pens without sprinkling devices, it was difficult to accurately distinguish between tail lesion scores 0 and 1; therefore, they were considered together. A minimum sample size of 50 pigs within each tail lesion score was selected. Data were collected from 101 pigs with tail lesions scored as 0-1, 51 pigs with tails scored as 2, 51 pigs with tails scored 3, and 66 pigs with tails scored 4. In all experimental days, pigs with the five different tail lesion scores were scored and imaged. All study pigs were imaged at the two locations (at the base of the tail and the ear).

### Capture and Analysis of Digital Thermography Images

The thermography camera was calibrated with Iron colors, ranging from 24°C and 37°C such that those areas on the skin surface of pigs with a temperature higher than the limit temperature appeared as yellow on the images. Accuracy of the infrared thermography camera was ±2^o^C. Emissivity (description of materials ability to emit energy by radiation) was set at 0.98 for all DTI as recommended by Soerensen et al. (38). The ambient temperature and humidity parameters were automatically recorded by the camera, together with the measured surface temperature. As all animals were lairaged in similar conditions, ambient temperature was not included in any correction formula. The distance between the equipment and the pig was targeted at ~1 m for all measurements, which was considered to be a safe distance for both animals and observers. All animals were imaged from ground level.

All images were downloaded and analyzed using the PC Software IRSoft for TESTO thermal images (v4.5). For each image, a circle area of ~10 cm of diameter was selected to highlight the hotspot (i.e., the highest temperature) in the selected area (Figure 1). The hotspot of the selected area for each DTI (base of the tail and the ear) was automatically displayed, in Celsius degree, on the thermal image markers of the software, yielding two values for each study pig.

### Statistical Analysis

Data were downloaded to Microsoft® Excel® for Mac OS where descriptive statistics were calculated. All other statistical analyses were conducted using SAS 9.3 (SAS Institute, Inc., Cary, NC, USA). The Shapiro test was used on the model residual information as well as the examination of the normal plot to evaluate the normal distribution.

The effect of tail lesion score and sex of the pig on the hotspot temperature of the selected infrared image areas, both at the base of the tail and the ear, were analyzed using linear mixed models (Proc Glimmix of SAS), using normal distribution. Tail lesion score and sex of the pig were included in the two models as fixed effects. For all models animal was considered as experimental unit. Interactions between tail lesion score and sex of the pig were removed from all models as they were not significant (*P* > 0.10). Results are reported as least square means ± standard error (S.E.) and statistic effects were reported when *P* ≤ 0.05. Finally, Pearson's correlation coefficient was used to analyze the relationship between the hotspot temperature of the selected infrared image area of the base of the tail and the ear of the pigs.

## Results

**Figure 3:**
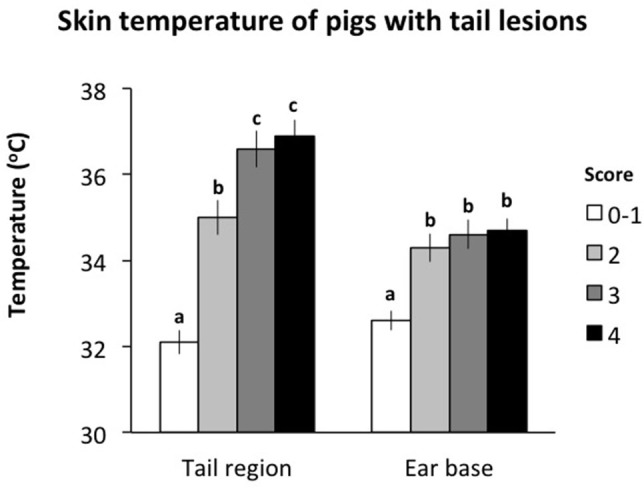
Skin temperature at the base of the tail and the ear of pigs within different tail lesion severity measured in the lairage. Different letters (a, b, c) represent significant differences (*P* < 0.05) between tail lesion scores.

There was an effect of the severity of tail lesion score on the skin temperature measured at the base of the tail and the ear (Figure 3). Skin temperature measured at the base of the tail was significantly lower for tails scored 0–1 than for all other tail lesion scores (*P* < 0.05). Pigs with tail lesions scored 2 had significantly lower skin temperatures at the base of the tail than pigs with tail lesions scored 3 or 4 (*P* < 0.05) while there was no difference in skin temperature at the base of the tail between pigs with tail lesion scores 3 and 4 (*P* > 0.05). Skin temperature measured at the ear base was significantly lower for tails scored 0–1 than for all other tail lesion scores (*P* < 0.05) with no difference between the other scores (*P* > 0.05). Sex did not affect the skin temperature of pigs recorded at the base of the tail and the ear (*P* > 0.05). Furthermore, there was a positive association between both measurements (*r* = 0.50; *P* < 0.001), such that increasing tail lesion severity was associated with increases in skin temperature measured at the base of the tail and the ear.

## Discussion

The aim of the current study was to evaluate potential differences in skin temperature of tail bitten pigs compared to pigs with intact tails measured at the base of the tail and of the ear by infrared thermography camera. The study also evaluated the association between temperatures recorded at the two locations. The present study confirms the hypothesis that increasing severity of tail damage is associated with an increase in skin temperature at both regions. As far as we know, this is the first study to investigate the skin temperature of pigs with tail lesions of differing severity.

Less severe tail injuries caused by tail biting are more visible on the carcass than in live animals (39–42). Indeed Carroll et al. (42) showed that tail lesions of every severity category became more visible after scalding and dehairing due to the removal of dirt and hair. However, we were interested in associations with body temperature and hence had to work with live animals. While the normal body temperature range in resting pigs varies between 37.0 and 39.6°C (30) and the normal rectal temperature in the thermoneutral zone is 39.3°C (34), the temperature of the inside surface of the pig ear ranges from 27.3 to 35.0°C, with a mean of 31.0±1.32°C (33). In pigs, temperature at the ear base is considered to give the best correlation between skin and rectal temperatures (34). Measured by a needle probe inserted under the skin, Soerensen et al. (38) reported 34.8°C as the highest temperature measured on the ear base of a living sow. In the current study, the average skin temperature at the ear base was 34.3±0.33 (score 2), 34.6±0.34 (score 3) and 34.7±0.28°C (score 4). These are similar to the values reported by Soerensen et al. (38) in healthy sows under experimental conditions. We expected to find higher skin temperatures in our study pigs due to the severity of the tail lesions; but it is important to highlight that measurements were taken after transportation from the farm and that the pigs were lairaged in pens with open walls such that the pigs were exposed to the weather. Unfortunately, the ambient temperature and humidity parameters in the lairage were not recorded in each experimental day, but the thermography camera automatically adjusted these data when measuring the surface temperature. On the other hand, to minimize the effect of the experimental day and the ambient temperature on the skin temperature of the pigs, pigs with the five different tail lesion scores were scored and imaged in all experimental days.

In the present study, there was an association between tail lesion severity and the skin temperature, such that increasing tail lesion score was associated with an increase in skin temperature at both the tail and ear base. This association is in agreement with Heinonen et al. (8) who found that the systemic inflammatory response seemed to be more severe in animals with deep tail inflammation. Tail biting induces a histopathologically detectable inflammation of the tail-end in slaughter pigs and leads to the spread of infection (8). Therefore, in the present study, it is suggested that the higher values of skin temperature at the tail base indicate signs of inflammation and potential infection (8), explaining the association between severity of tail lesion and the skin temperature at the base of the tail.

Previous studies showed that infection from the tail area to the sacral vertebrae via the lymph or blood stream causes pyemic foci in other parts of the body, especially lungs and vertebrae (43). Tail bitten pigs show higher serum protein concentrations and a higher prevalence of anemia than uninjured pigs (6), indicating that victims of tail biting are more severely challenged by (bacterial) infections (44) and chronic inflammation (45), finally showing the elevated systemic temperatures. Therefore, tail wounds from biting appear to cause infection elsewhere in the body (22). Unfortunately we did not measure either local or general infection and, therefore, such effect warrants further research. Furthermore, despite our study did not include physiological parameters, Warris et al. (33) also reported a positive correlation between the temperature of the inside surface of the ear and the activity of serum creatine kinase, as well as between concentrations of serum cortisol and blood temperature, suggesting that the hotter pigs were suffering from higher levels of stress. Stress (46, 47) and inflammation (48) cause an increase in body temperature. Stress (49) and fever (36) significantly elevate the skin temperature in pigs, although with a delay compared to rectal temperature. Schmidt et al. (50) were able to detect febrile sows from eye and ear base temperatures using infrared thermography images. Increases in skin temperature were also reported in pigs with palpable symptoms of osteoarthrosis (51) and with high blood temperature at exsanguination (33).

From the current study, it is important to highlight that pigs with moderate tail lesions (score 2) showed a significantly higher skin temperature compared to pigs with intact tails or mild tail lesions. This finding suggests that even pigs with moderate tail lesions (score 2) may have general inflammation and infection. This is in agreement with Smith and Penny (52) who suggest that even tail damage restricted to puncture wounds can readily set up pyemia. The Scientific Report of EFSA (4) highlighted the importance of recording both moderate and severe tail damage at meat inspection. Previous studies showed that pigs with even moderate tail lesions have higher odds of having abscesses or pleurisy/embolic pneumonia (21), pathological lesions, carcass trimmings/condemnations and reduced carcass weight (12, 20, 22, 24). Moreover, even for carcasses without any disease potentially detected at post-mortem inspection, Teixeira et al. (22) reported a reduction in carcass weight associated with tail lesions scored as moderate or severe (scores > 2).

Finally, the findings from the present study support that the infrared thermography camera may be used as a Precision Livestock Farming (PLF) tool for recognizing/detecting tail lesions of different severity in live pigs, with high value to the farmer in managing tail biting on farm, especially in undocked pigs.

## Conclusion

This study showed that the severity of tail lesions increases the skin temperature of pigs measured at the base of the tail or the ear. The findings suggest that even pigs with moderate tail lesions (score 2) may have general inflammation and infection, evidenced by the elevated systemic temperature compared to pigs with none or mild tail lesion (score 0-1), which provides further support for the importance of more detailed measurements of tail lesion severity in pigs.

## Data Availability Statement

The datasets generated for this study are available on request to the corresponding author.

## Ethics Statement

This study was part of a research project approved by the Scientific Ethics Committee for Animals and Environmental Care of the Pontificia Universidad Católica de Chile.

## Author Contributions

DT, LB, and DE-H contributed to the concept of the work and interpreted data. DT and DE-H initiated, performed statistical analysis, and designed the study. DT performed the experiment and wrote the manuscript. LB and DE-H contributed to the manuscript. All authors approved the final version of the manuscript.

## Conflict of Interest

LB was employed by the company Pig Development Department, Moorepark, TEAGASC. The remaining authors declare that the research was conducted in the absence of any commercial or financial relationships that could be construed as a potential conflict of interest.
